# How education level affects postoperative rehabilitation and follow-up: a single-center experience

**DOI:** 10.1186/s12894-023-01282-x

**Published:** 2023-07-18

**Authors:** Jin Ji, Yuchen Yang, Zeyu Chen, Wenhui Zhang, Shaoqin Jiang, Xianqi Shen, Jili Zhang, Lu Lin, Min Qu, Yan Wang, Xu Gao

**Affiliations:** 1 Department of Urology, Changhai Hospital, Navy Medical University (Second Military Medical University), 168 Changhai Road, Shanghai, 200433 P. R. China; 2 Department of Urology, Naval Medical Center, Naval Medical University, Shanghai, China; 3 Nursing Department, Naval Hospital of Eastern Theater Command, PLA, Zhoushan, China; 4 Department of Urology, The Second Affiliated Hospital of Soochow University, Jiangsu, China; 5 Department of Urology, Fujian Union Hospital, Fujian Medical University, Fuzhou, Fujian China

**Keywords:** Radical prostatectomy, Education level, Postoperative recovery, Follow-up, Pc-follow

## Abstract

**Background:**

Radical prostatectomy remains the fundamental treatment for prostate cancer, and improving patients’ compliance with postoperative follow-ups is essential for improving patients’ quality of life. This study investigates the effect of education levels on patients’ recovery and follow-up after radical prostatectomy.

**Methods:**

Data from 1,112 patients undergoing radical prostatectomy between 2011 and 2020 were collected using medical records, and “pc-follow” systems were used to collect patients’ baseline information, education level, pathological information, number of outpatient visits, the time interval between each visit, and PSA test data.

**Results:**

Regarding postoperative outpatient data, there was no difference in the number of outpatient visits among the different education level groups in Shanghai (*P* = 0.063). A significant difference was found in the interval between outpatient visits among the groups (*P* < 0.001). Furthermore, significant differences were detected in the number and duration of outpatient clinic visits among the education level groups in all patients (*P* = 0.016, *P* = 0.0027). By contrast, no significant difference was found in the recovery time of urinary continence between all patients and those in Shanghai, grouped according to education level (*P* = 0.082, *P* = 0.68). For all patients and patients in the Shanghai area, the number of PSA follow-ups increased gradually with an increasing level of education (*P* < 0.001, *P* = 0.0029).

**Conclusions:**

Education level affected the number of postoperative clinic visits, compliance, and the number of PSA tests. However, no significant effect on the recovery of urinary continence was found. Further, clinicians must increase their focus on patients with low education levels to achieve equitable access to health services for all patients.

**Supplementary Information:**

The online version contains supplementary material available at 10.1186/s12894-023-01282-x.

## Background

Globally, prostate cancer (PCa) is a common malignant tumor of the male urinary system [1], and it has been reported that the incidence of PCa has increased rapidly in China [2]. Radical prostatectomy is becoming the preferred approach in the surgical management of prostate cancer. It effectively prolongs patients’ survival time, but several issues remain. Patients who underwent radical prostatectomy for PCa have consistently reported complications (urinary incontinence and erectile dysfunction), severely affecting their quality of life [3, 4], with some exhibiting biochemical recurrence [3]. Periodic reexamination is effective for preventing or promptly detecting and diagnosing postoperative complications, and postoperative efficacy, recurrence, or metastasis can be evaluated by biochemical tests [4, 5]. Improving patients’ compliance with postoperative follow-ups is essential to improving patients’ quality of life after surgery [6].

Several studies have investigated the effect of a patient’s socioeconomic status (SES) on prostate cancer outcomes. Education level has been used as the most specific indicator of SES [7–9]. Research has established a correlation between higher socioeconomic status and better cancer outcomes after prostate cancer [7]. In addition, previous studies have shown the influence of a patient’s educational level on treatment selection for newly diagnosed prostate cancer [8, 10]. However, little data is available on the relationship between patients’ academic level after radical prostatectomy and postoperative follow-up and recurrence.

Since education level significantly impacts the patient’s lifestyle, we hypothesized that patients with higher educational levels would recover faster after surgery and have better tumor prognosis. Therefore, our study investigated the relationship between relapsed patients’ educational levels after radical prostatectomy and postoperative follow-up. We also examined the differences in postoperative visit timeliness and adherence among patients with varying academic levels.

## Methods

### Study design

Information was collected on all patients who underwent radical prostatectomy in the same attending group from 2011 to March 23, 2020. All patients signed consent forms. The Medical Ethics Committee of Changhai Hospital approved the study (No. CHEC2022-046), which was conducted according to all tenets of the Declaration of Helsinki.

### Data collection

We collected patient data from the medical record system and PC-Follow database. The obtained baseline information included the patient’s height, weight, age, education information, and place of residence. Pathology information included pathological staging, bilateral lymph node biopsy, prostate capsule invasion, whether the resection margin was positive, seminal vesicles and nerve invasion, Gleason score, and vascular invasion. Surgery information included operation time, operation method, and lymph node dissection or not.

Follow-up information included the last visit, outpatient visits and their number, postoperative PSA, the date of biochemical recurrence, endocrine therapy status, and whether it has progressed to CRPC.

### Education level

All patients were divided into four groups for analysis according to their education levels. The first group consisted of patients with no formal education. The second group was individuals with elementary school and junior high school educations. The third group comprised patients with high school and technical secondary school educations. The fourth group was those with college degrees and above.

### Statistical analysis

Data were analyzed using SPSS software Version 21.0 (IBM) and Prism GraphPad 7.0 (GraphPad Software). Age and BMI are described by mean (SD). The age and BMI of each group were analyzed using one-way ANOVA. The patient’s PSA is described by median (IQR). The Kruskal–Wallis test examined PSA and fPSA at different education levels. For data with statistical differences, the Bonferroni method was used to compare between two groups. Gleason score, pT stage, pN stage, and M stage were analyzed using Kruskal–Wallis’s method. The Chi-square analyzed capsule invasion, nerve invasion, seminal vesical invasion, and positive surgical margin in different education level groups. The association between education levels and the number of outpatient visits, the interval between outpatient visits, and the recovery time of urinary control were analyzed using the Kruskal–Wallis test.

## Results

The study included 1,112 patients after radical prostatectomy. The characteristics of these patients are summarized in Table 1. All patients were divided into four groups according to their education level. Group 1 was patients with no formal education; Group 2 was primary school and middle school education; Group 3 was senior high school or technical secondary school education; Group 4 was university or college education. The average patient age was 66.77 (SD: 6.86) years, and the average BMI was 24.57 (SD: 2.96). The average preoperative PSA was 22.82 ng/ml, and fPSA was 2.896 ng/ml. Table 1 summarizes patients’ pathological characteristics, including Gleason score, pT stage, metastasis, capsule, lymphatic, nerve, and seminal vesicle invasion, and whether the resection margin was positive.

**Table 1:** Characteristics of patients

	All(1112)	Group1(33)	Group2(442)	Group3(237)	Group4(384)	*P*-value
Age(mean, sd)	66.7(6.86)	68.97(5.07)	67.4 (5.89)	66.9 (7.13)	65.73 (7.76)	*P* < 0.001※
BMI(mean, sd)	24.57(2.96)	24.42(2.96)	24.68(2.95)	24.4(2.84)	25.57(2.97)	*P* = 0.68
PSA(mean,IQR)	22.82(8.439–26.78)	27.43(7.932–32.18)	26.16(8.853–33.48)	21.75(8.998–25.58)	19.4(7.94–21.52)	*P* = 0.0018※
fPSA(mean, IQR)	2.896(0.869–2.901)	2.889(0.7075–4.474)	3.142(0.94–3.41)	2.44(0.94–3.41)	2.921(0.8005–2.294)	*P* = 0.0085※
Gleason score						*P* = 0.2373
≤ 6	105	2	38	22	42	
7	616	15	252	145	199	
≥ 8	313	13	113	62	118	
pT stage						*P* = 0.5105
≤ 2a	173	6	58	39	68	
2b	56	3	22	13	17	
≥ 2c	893	24	361	185	297	
pN stage						*P* = 0.5073
pNx/pN0	998	28	398	211	350	
pN1	109	5	44	26	32	
M stage						*P* = 0.8585
M0/Mx	1101	33	437	234	381	
M1	11	0	5	3	3	
Capusle invasion						*P* = 0.0149※
Positive	513	16	224	118	154	
Negative	599	17	218	119	230	
Nerve invasion						*P* = 0.3975
Positive	652	18	278	141	215	
Negative	417	15	157	93	151	
Seminal vesical invasion						*P* = 0.3294
Positive	209	8	93	44	61	
Negative	867	25	343	192	306	
Surgical margin						*P* = 0.31
Positive	473	15	206	103	149	
Negative	596	18	229	131	217	

AGE, BMI one way ANOVA, PSA, fPSA, Gleason score, pT stage, pN stage, M stage Kruskal–Wallis test Capsule invasion, Nerve invasion, Seminal vesical invasion, Positive surgical margin Chi-square

Sixteen patients education level information is not available

※ statistical signifiance

**Fig. 1:**
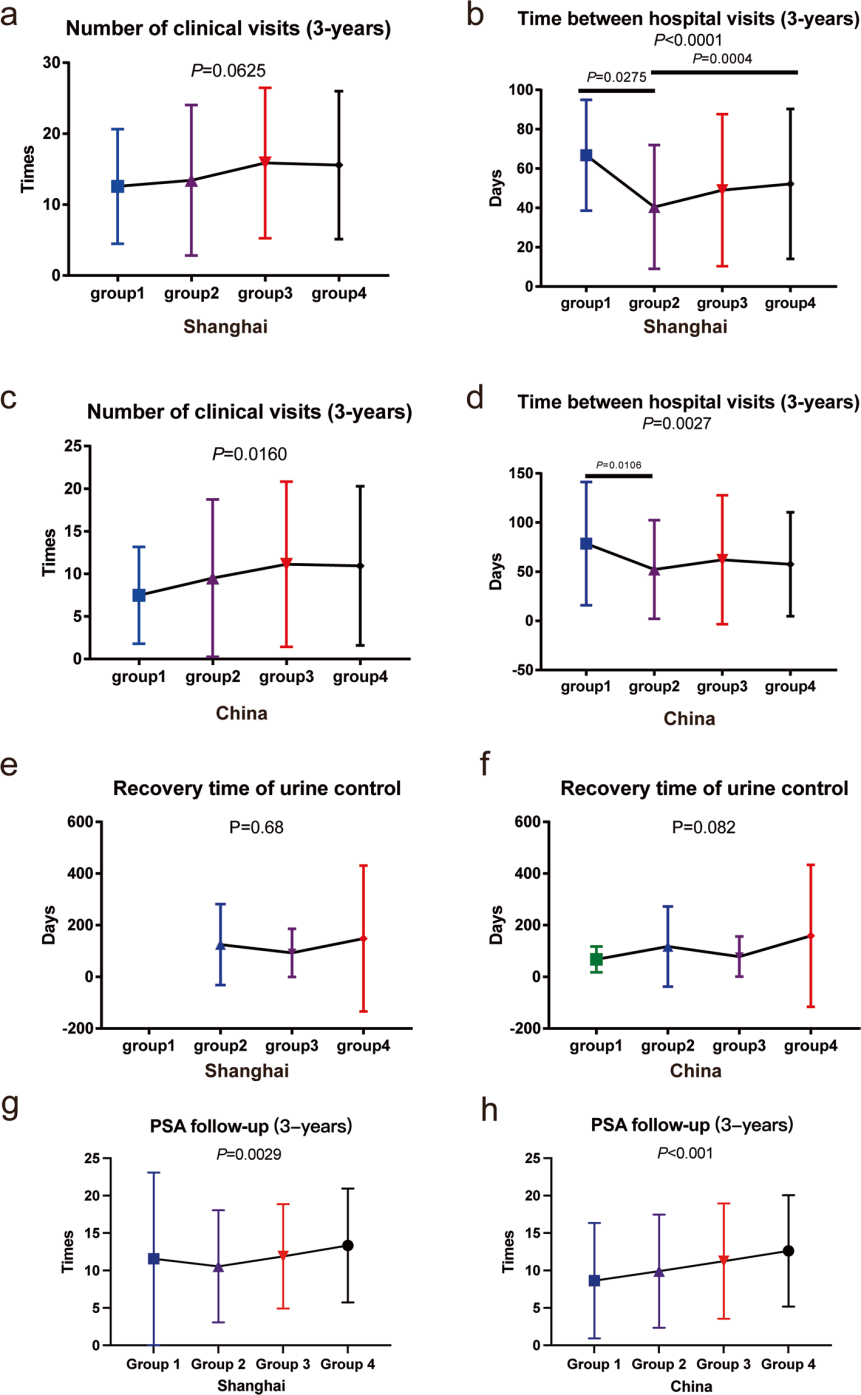
Effect of education level on postoperative rehabilitation and follow-up of patients. Impact of education level on the number of postoperative outpatient visits in China (**c**) and Shanghai (**a**). Influence of education level on the interval between postoperative outpatient visits in Chinese (**d**) and Shanghai patients (**b**). Effect of education level on postoperative continence recovery time in Chinese (**f**) and Shanghai (**e**) patients. Influence of education level on the number of postoperative PSA tests in Chinese (**h**) and Shanghai patients (**g**)

We obtained data from the hospital in Shanghai. Therefore, we investigated the postoperative outpatient status of all 453 patients in that area. We excluded patients who visited the outpatient clinic less than two times after surgery because they were no longer in this hospital after surgery. We then investigated the postoperative visits of a total of 425 patients. Groups 1, 2, 3, and 4 included 7, 166, 99, and 153 patients, respectively. The average number of outpatient visits of Shanghai patients over three years was 14.75 (SD: 10.53). No significant difference was found in the number of postoperative outpatient visits in Shanghai patients (Fig. 1a). Education level did not affect the number of patient visits (*P* = 0.0625). However, significant differences were found in the groups in the interval between outpatient visits of patients in Shanghai (*P* < 0.0001, Fig. 1b). Additionally, we found that Groups 1 and 4 had more extended hospital visits interval than Group 3 (Fig. 1b, *P* = 0.0275; *P* = 0.0004).

Next, we studied the circumstances of patients from all over the country (including Shanghai). We excluded patients who did not continue to visit a doctor in this hospital after the operation, leaving 879 enrolled cases. The average number of hospital visits for all patients within three years was 10.25 (SD: 9.287), and the interval between visits was 57.02 (SD:55.21) days. Education level was found to significantly impact the number of outpatient visits (Fig. 1c, *P* = 0.016), whereby an improvement in education level was correlated with a gradual increase in outpatient clinics. Moreover, the education level also significantly impacted the interval between outpatient visits (Fig. 1d, *P* = 0.0027). The time between outpatient visits in Group 4 was considerably higher than that in Group 2 (*P* = 0.0106).

Subsequently, we focused on the impact of educational level on urination recovery time. Education level did not affect the recovery time of urination control in Shanghai (Fig. 1e, *P *= 0.68) or the whole of China (Fig. 1f, *P* = 0.082). Through analyzing the number of PSA tests uploaded by patients and doctors through the pc-follow database, we found that with the improvement of education level, the number of PSA tests increased in patients in Shanghai (Fig. 1g, *P* = 0.0029) and the whole country (Fig. 1h, *P* < 0.001). Further, a higher educational level was not found to shorten the interval between visits for relapsed patients in Shanghai or the whole country (Figure S1a, b).

## Discussion

Radical prostatectomy is widely used to treat prostate cancer. However, the associated complications impacting patients’ quality of life cannot be ignored [11]. Patients should be strictly followed-up and reviewed regularly to detect recurrence early on and to improve complications [12]. This strategy calls for prolonged monitoring and chronic-care management by healthcare professionals to help patients after radical prostatectomy learn about their condition and improve their quality of life when living with it [13]. A study by Froehner et al. showed that patients with higher education levels had lower mortality after radical prostatectomy [14]. Education level can be used as an independent prognostic indicator and a reference factor for treatment selection. People with higher education levels have a stronger desire to be screened for and learn about cancer [15]. Tomic et al. reported lower 6-year mortality in PCa patients with high SES [7]. Zheng et al. demonstrated that patients with HCC (Hepatocellular carcinoma) with low educational levels had a poor prognosis [16], and many studies have shared similar opinions [17–19].

Postoperative urinary continence is one of the most critical indicators of life quality and treatment satisfaction [20, 21]. We took the date of the first visit to the outpatient clinic after urinary control returned minus the date of surgery as the recovery time. During this period, patients may have recovered but not followed up in time, resulting in prolonged recovery time of urinary control. Our mean time to recover urinary control after surgery was longer than in other studies [22]. Nevertheless, we did demonstrate that education level did not affect the time to recover urinary control.

We have focused on the impact of patients’ education level on cancer prognosis and the relationship between patients’ education level after radical prostatectomy and postoperative follow-up. We divided all patients from the whole country (including Shanghai) into four groups according to their education level and found a significant impact of education level on the interval between outpatient visits among all groups. In addition, the more highly-educated patients made several post-treatment visits. However, education level did not affect the recovery time of urination control in Shanghai or China in general.

The current study suggests that although the prognosis may be uneven across different patient groups, patients with lower education levels require specialized attention. Considerable variability exists in the economic, educational, and medical distribution throughout the whole country. China’s sizeable agricultural population suggests the need to improve this group’s postoperative visit timeliness and adherence [23, 24]. During the COVID-19 pandemic, more attention should be paid to the follow-up of patients after surgery, especially for low-income groups [25]. Li et al. reported that patients with low educational levels were likelier to receive treatment-related information [26]. Li et al. reported that adequate pre-chemotherapy patient education in colorectal cancer patients reduced anxiety regarding chemotherapy [27]. This finding prompts us to pay more attention to patients with lower educational levels.

While guiding the follow-up of patients, we should pay more attention to the recovery of postoperative complications. As a result of sufficient patient education, such as distributing urinary continence recovery manuals, making WeChat mini-programs, and rehabilitation training videos, no significant difference was found in the urinary continence recovery time between groups of different education levels. With the rapid development of the internet and information technology, using more digital health tools to educate patients can gradually reduce the inequality of access to medical consultations caused by education levels [28, 29]. Changhai Hospital also vigorously promotes the pc-follow database in WeChat access [30]. The database aims to make it easier for patients to upload postoperative examination results in different hospitals to establish a closer connection between doctors and patients. However, patients with higher education levels have increased postoperative PSA frequency, which reflects the need to strengthen patient follow-up guidance further. This conclusion is similar to that in Seikkula’s previous study, which found that patients with higher education levels had more PSA testing [31]. For patients with lower education levels, proactive telephone, and Internet follow-up were used to understand their postoperative recovery, resolve their disease questions, and provide personalized guidance.

One limitation of our study is the patients’ follow-up period. We analyzed the outpatient visits after radical prostatectomy within three years. Postoperative outpatients should receive extended monitoring to provide more supporting evidence.

Another limitation is that all cases were from the same institution in Shanghai; among the 1,112 patients included in the study, 659 were from regions outside of Shanghai. Therefore, face-to-face follow-up was challenging for some patients, and they could not keep their appointments.

## Conclusions

Our research indicates that education level significantly impacts patients after radical prostatectomy regarding the timeliness and compliance of postoperative follow-up visits. For patients with low education levels, it is necessary to conduct long-term monitoring and postoperative follow-ups. Future efforts could pay close attention to patients’ education levels to provide personalized treatments and healthcare.

## Supplementary Information

**Additional file 1:** **Figure S1. **Effect of education level on the interval between visits for relapsed patients in Shanghaiand whole country.

## Data Availability

The raw data supporting the conclusions of this article will be made available by the authors without undue reservation. Please contact Prof. Xu Gao for the data of the study.
