# Supplementary material for: How education level affects postoperative rehabilitation and follow-up: a single-center experience
Source: BMC Urol. 2023 Jul 18;23:123. doi: 10.1186/s12894-023-01282-x (PMC10354960; doi:10.1186/s12894-023-01282-x)
Supplement: Supplementary file 1 — Additional file 1: Figure S1. Effect of education level on the interval between visits for relapsed patients in Shanghaiand whole country. [file 12894_2023_1282_MOESM1_ESM.docx]

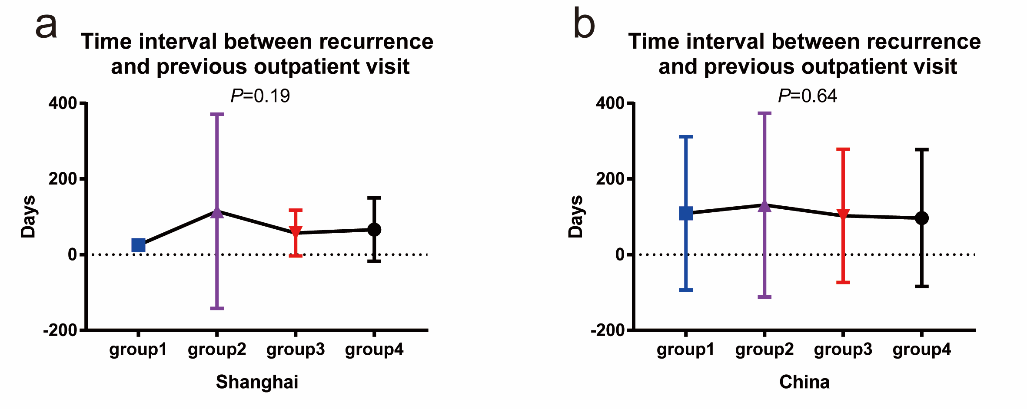


**Figure S1** Effect of education level on the interval between visits for relapsed patients in Shanghai(**a)** and whole country(**b**).
